# Distal radius plate of CFR-PEEK has minimal effect compared to titanium plates on bone parameters in high-resolution peripheral quantitative computed tomography: a pilot study

**DOI:** 10.1186/s12880-017-0190-z

**Published:** 2017-02-27

**Authors:** Joost J. A. de Jong, Arno Lataster, Bert van Rietbergen, Jacobus J. Arts, Piet P. Geusens, Joop P. W. van den Bergh, Paul C. Willems

**Affiliations:** 1 NUTRIM School for Nutrition and Translational Research in Metabolism, Maastricht University, Maastricht, The Netherlands; 2 Department of Rheumatology, Maastricht University Medical Center, Maastricht, The Netherlands; 3 Department of Anatomy and Embryology, Maastricht University, Maastricht, The Netherlands; 4 Faculty of Biomedical Engineering, Eindhoven University of Technology, Eindhoven, The Netherlands; 5 Department of Orthopedic Surgery, Maastricht University Medical Center, Maastricht, The Netherlands; 6 CAPHRI School for Public Health and Primary Care, Maastricht University, Maastricht, The Netherlands; 7 Faculty of Medicine and Life Sciences, Hasselt University, Hasselt, Belgium; 8 Department of Internal Medicine, VieCuri Medical Center, Venlo, The Netherlands

**Keywords:** Injury/fracture healing, HRpQCT, Implant, Distal radius, CFR-PEEK

## Abstract

**Background:**

Carbon-fiber-reinforced poly-ether-ether-ketone (CFR-PEEK) has superior radiolucency compared to other orthopedic implant materials, e.g. titanium or stainless steel, thus allowing metal-artifact-free postoperative monitoring by computed tomography (CT). Recently, high-resolution peripheral quantitative CT (HRpQCT) proved to be a promising technique to monitor the recovery of volumetric bone mineral density (vBMD), micro-architecture and biomechanical parameters in stable conservatively treated distal radius fractures. When using HRpQCT to monitor unstable distal radius fractures that require volar distal radius plating for fixation, radiolucent CFR-PEEK plates may be a better alternative to currently used titanium plates to allow for reliable assessment. In this pilot study, we assessed the effect of a volar distal radius plate made from CFR-PEEK on bone parameters obtained from HRpQCT in comparison to two titanium plates.

**Methods:**

Plates were instrumented in separate cadaveric human fore-arms (n = 3). After instrumentation and after removal of the plates duplicate HRpQCT scans were made of the region covered by the plate. HRpQCT images were visually checked for artifacts. vBMD, micro-architectural and biomechanical parameters were calculated, and compared between the uninstrumented and instrumented radii.

**Results:**

No visible image artifacts were observed in the CFR-PEEK plate instrumented radius, and errors in bone parameters ranged from −3.2 to 2.6%. In the radii instrumented with the titanium plates, severe image artifacts were observed and errors in bone parameters ranged between −30.2 and 67.0%.

**Conclusions:**

We recommend using CFR-PEEK plates in longitudinal in vivo studies that monitor the healing process of unstable distal radius fractures treated operatively by plating or bone graft ingrowth.

## Background

Carbon-fiber-reinforced poly-ether-ether-ketone (CFR-PEEK) is increasingly being used as a material for orthopedic implants, e.g. in spinal cages, hip prostheses, or intramedullary nails [1–4]. One of the main advantages of this material is that CFR-PEEK has been found to have superior radiolucency as compared to other materials that are conventionally used for orthopedic implants, such as stainless steel or titanium. Therefore, implants made out of CFR-PEEK allow metal-artifact-free postoperative monitoring by computed tomography (CT), magnetic resonance imaging (MRI) and radiographs [5].

Recently, it has been shown that the repair process of stable distal radius fractures can be monitored longitudinally in vivo using high-resolution peripheral computed tomography (HRpQCT) [6, 7]. For clinical and research purposes, it would be of great interest to monitor the repair process of unstable fractures or the ingrowth of bone graft or bone graft substitutes after corrective osteotomy as well. Whereas stable distal radius fractures are usually treated by immobilization with a fiberglass cast, which only has a limited effect on the bone parameters obtained by HRpQCT [8], volar distal radius plates (VDRPs) as internal fixation are a generally accepted treatment in case of unstable distal radius fractures or after corrective osteotomy [9]. Since VDRPs are usually made from stainless steel or, more common, from titanium, they cause severe artifacts on CT and HRpQCT images [10], including scattering, beam hardening and streak occurrence [11]. Besides reduced sensitivity of CT measurements, this may result in an over- or underestimation of the bone density, micro-architectural and biomechanical parameters that are derived from such images [10].

Besides other implants, VDRPs made of CFR-PEEK were recently developed. Whereas it is known that the polymers alone will not cause major artifacts in clinical CT scans, potentially they could affect the density and morphology measurements in HRpQCT images due to changes in beam hardening or scatter-related noise. Also, such plates typically contain metal fibers or other additions to increase their visibility on radiographs that may cause artifacts. It therefore is unknown whether the quantitatively obtained bone parameters obtained from HRpQCT images remain unaffected. We expect that a CFR-PEEK plate will lead to less artifacts in HRpQCT images as compared to metal plates, and thus might be a suitable alternative to the currently used titanium plates in studies monitoring the healing process of unstable fractures or ingrowth of bone graft or bone graft substitutes after corrective osteotomy.

Therefore, the aim of this study was to compare the effect of VDRPs made of titanium or made of CFR-PEEK on the occurrence of artifacts in HRpQCT images and on the volumetric bone mineral density (vBMD), micro-architectural and biomechanical parameters that are obtained from these images.

## Methods

### Volar distal radius plates

Three types of commercially available VDRPs from different firms were compared in this study. Plate #1 (Standard 5-hole Volar Distale Radius Plate, Icotec AG, Switzerland) had a thickness of 2.5 mm and consisted of CFR-PEEK to which 0.5% tantalum fibers were added for radiological visibility. Plate #2 (VariAx^TM^ Standard Anatomical Volar Distal Radius Plate, Strycker Corporation, United States of America) was 2 mm thick and made of titanium alloy Ti6Al4V, which contained 89% titanium, 6% aluminum and 4% vanadium. Plate# 3 (2.4 mm Variable Angle LCP Volar Extra-Articular Distal Radius Plate, Synthes AG, Switzerland) was 2.4 mm thick and also made of a titanium alloy, i.e. Ti6Al7Nb containing 86% titanium, 6% aluminum and 7% niobium.

### Fresh frozen fore-arms

For this study three intact human fore-arms (right side) were isolated from three cadavers. A handwritten and signed codicil from each donor, posed when still alive and well, is kept at the Department of Anatomy and Embryology, Faculty of Health, Medicine and Life Sciences, Maastricht University, Maastricht, The Netherlands. This is required by Dutch law for the use of cadavers for scientific research and education. The fore-arms were thawed for 8 h at 16 °C before surgery was started.

### Surgery

**Fig. 1:**
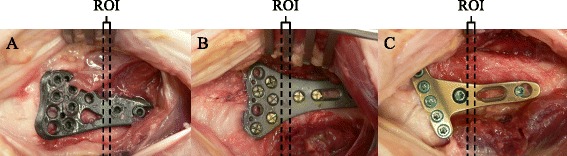
Each volar distal radius plate was instrumented in one cadaveric forearm by means of a standard volar approach. The CFR-PEEK plate is shown in panel **a** and the two titanium plates in panels **b** and **c**. In each plate, five screws were fixed in the metaphysis and two screws were fixed in the shaft. The region of interest (ROI, *between dotted lines*) was set such that it was fully covered by a part of the plate without screws or holes

Each plate was instrumented by an experienced orthopedic surgeon (PW) in one fore-arm by means of a standard volar approach and fixed to the distal radius according to the instruction manual provided by the manufacturer. During surgery, employees of the respective firms were present to support the surgeon and to make sure the proper surgical techniques were used. In all plates, five screws were fixed in the metaphysis distally from the region of interest (ROI) and two screws were fixed in the shaft proximally from the ROI (Fig. 1). The screws were made of the same material as the respective plate. The plates were removed after HRpQCT scans had been performed.

### HRpQCT scanning

**Fig. 2:**
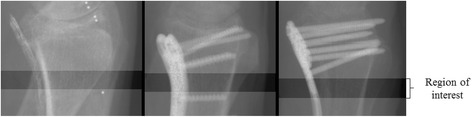
Lateral scoutviews of each instrumented radius showing the region of interest (ROI) in each radius. The ROI was chosen at a location where the radius was fully covered by a part of the plate without holes and that was not intersected by screws. The tantalum fibers in the CFR-PEEK plate (*left*) allow visualization of the plate

Both after instrumentation and after removal of the plates, the fore-arms were scanned twice, with repositioning between each scan (thus four times in total) by HRpQCT (XtremeCT, Scanco Medical AG, Switzerland) using clinical in vivo settings by the manufacturer (60 kVp effective energy, 900 μA tube current, and 100 ms integration time). A standard stack of 110 transverse images with an isotropic voxel size of 82 μm was acquired, covering a region of 9 mm in each fore-arm. The ROI was set at the distal end of the radius and which was covered by a part of the plate without screw holes (Fig. 2). Also, the ROI was chosen such that it was located in between the five distal and two proximal screws to eliminate distortion by the screws.

### Bone density and micro-architecture

For each forearm, the HRpQCT scans with and without a plate were matched by slice-matching to make sure the same region was analyzed. The HRpQCT images within the ROI were then evaluated using the standard patient evaluation protocol provided by the manufacturer and has been described earlier in detail elsewhere [12]. In short, after contouring of the periosteal boundary of the radius, the total region was separated into a cortical and trabecular region. For each region, the volumetric bone mineral density (Dtot, Dcort and Dtrab, respectively) [mgHA/cm^3^] was assessed. The mineralized bone was extracted and a segmented image was created [12] from which the following micro-architectural parameters were assessed: trabecular number (Tb.N) [mm^−1^], thickness (Tb.Th) [mm] and separation (Tb.Sp) [mm]. For the cortical region, the thickness (Ct.Th) [mm] was calculated [12].

### Micro finite element analysis

μFE models were created directly from the segmented HRpQCT images, similar to earlier studies [13, 14]. In short, each voxel representing bone was converted into a brick element of the size, thus creating a representative μFE model of the bone’s micro-architecture. Typically, these μFE models consisted of 3 to 4 million elements. Equal material properties were assigned to every element, i.e. a Young’s modulus of 10 GPa and a Poisson ratio of 0.3 [14]. By applying a ‘high friction’ compression test in the axial direction as described by Pistoia et al., the following biomechanical parameters were estimated: stiffness (Scomp) [kN/mm], which can be described as the resistance against displacement; and ultimate failure load (F.Ult) [kN], which is the load at which the fracture criterion is met, e.g. the strain in at least 2% of the volume exceeds 0.7% [13].

### Statistics

Reproducibility of the bone density, micro-architectural and biomechanical parameters was expressed using the root mean-square coefficient of variation (RMSCV%) [15]:

1 $$ \mathrm{RMSCV}\%=\sqrt{\frac{{\mathrm{d}}^2}{2}}/\overline{\mathrm{x}}\cdot 100\% $$

with *d* the difference between the first and second measurement, and $\overline{x}$ the mean of the two measurements.

Means for the bone parameters in each instrumented and uninstrumented radius were calculated from the duplicate measurements. The errors in the bone parameters introduced by each plate were expressed as percent differences between the instrumented and uninstrumented radius, with the uninstrumented radius as 100% reference.

## Results

### HRpQCT images

**Fig. 3:**
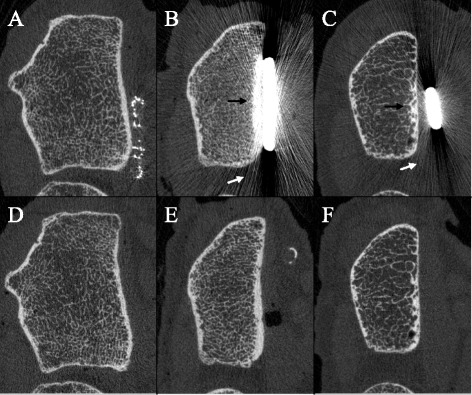
Representative HRpQCT slices in each radius with (**a**, **b** and **c**) and without instrumented plate (**d**, **e** and **f**). The CFR-PEEK plate with tantalum fibers (panel **a**) caused less visible image artifacts as compared to the titanium plates (panel **b** and **c**), which caused streak artifacts (*white arrows*) and an increased intensity of voxels close to the titanium plates (*black arrows*)

HRpQCT images of each radius with and without plate are shown in Fig. 3. The tantalum fibers that were incorporated into the CFR-PEEK plates were clearly visible, and no visible image artifacts were observed when the CFR-PEEK plate was instrumented (Fig. 3a). In the radii instrumented with the titanium plates, severe image artifacts were seen (Fig. 3b and c). These image artifacts consisted of streaks and a higher intensity of the bone voxels that are closely located to the plate.

### Errors in bone parameters

The reproducibility (RMSCV%) of the bone density, micro-architectural and biomechanical parameters ranged from 0.0 to 2.3% (Table 1).

**Table 1:** Reproducibility (RMSCV%) calculated from duplicate HRpQCT measurements at the uninstrumented and instrumented radii

Bone parameter	RMSCV% Radius #1		RMSCV% Radius #2		RMSCV% Radius #3	
	Un-instrumented	Instrumented (CFR-PEEK)	Un-instrumented	Instrumented (Titanium)	Un-instrumented	Instrumented (Titanium)
vBMD						
Dtot	0.52	2.18	0.02	1.29	0.00	0.20
Dtrab	0.10	1.95	0.34	0.00	0.31	0.62
Dcort	0.31	1.48	0.24	1.13	0.01	0.06
Micro − architecture						
BV/TV	0.00	1.76	0.47	0.00	1.25	0.00
Tb.N	2.24	0.92	0.00	1.19	0.72	0.67
Tb.Th	1.84	0.90	0.00	1.66	0.00	1.11
Tb.Sp	2.16	0.99	0.16	1.13	0.37	0.08
Ct.Th	1.50	1.55	0.00	1.65	0.93	0.00
Biomechanical						
Scomp	0.42	1.38	0.28	1.71	0.02	0.49
F.Ult	0.28	1.30	0.40	2.23	0.06	0.54

Abbreviations: *vBMD* volumetric bone mineral density, *Dtot* total density, *Dtrab* trabecular density, *Dcort* cortical density, *BV/TV* bone to total volume, *Tb.N* trabecular number, *Tb.Th* trabecular thickness, *Tb.Sp* trabecular separation, *Ct.Th* cortical thickness, *Scomp* compression stiffness, *F.Ult* ultimate failure load

In Table 2, the bone parameters measured per uninstrumented and instrumented distal radius are shown, together with the percent errors that were introduced by each plate. All errors in bone parameters are in comparison to the same but uninstrumented radius. For the radius instrumented with the CFR-PEEK plate, percent errors in bone parameters ranged from −3.2% in Ct.Th to +2.6% in Tb.Sp, and the smallest error was observed in Dtrab, with −0.8%. For the radii instrumented with the titanium plates, in general the percent errors were larger. For the radius instrumented with plate #2, the percent errors in bone parameters ranged from −43.2% in Tb.Sp to +67.2% in Dtrab, and the smallest error was observed in Scomp, with +7.5%. For the radius instrumented with plate #3, the largest percent errors in bone parameters were also found in Tb.Sp and Dtrab, being −11.2% and +30.2%, respectively. The smallest error was observed in F.Ult, with +0.1%.

**Table 2:** Bone mineral density, micro-architectural and biomechanical parameters measured with HRpQCT at the uninstrumented and instrumented distal radius and the percent difference between them

Bone parameter	Radius #1			Radius #2			Radius #3		
	Un-instrumented	Instrumented (CFR-PEEK)	Error (Δ%)	Un-instrumented	Instrumented (Titanium)	Error (Δ%)	Un-instrumented	Instrumented (Titanium)	Error (Δ%)
vBMD									
Dtot [mgHA/cm^3^]	273	266	(−2.5)	343	520	(+51.8)	229	245	(+6.7)
Dtrab [mgHA/cm^3^]	146	145	(−0.8)	181	304	(+67.2)	62	80	(+30.2)
Dcort [mgHA/cm^3^]	862	848	(−1.6)	839	1004	(+19.6)	853	850	(−0.3)
Micro − architecture									
BV/TV [−]	0.122	0.121	(−1.2)	0.152	0.253	(+67.0)	0.052	0.067	(+30.1)
Tb.N [mm^−1^]	1.58	1.54	(−2.2)	1.92	2.98	(+55.0)	0.96	1.06	(+10.5)
Tb.Th [mm]	0.077	0.079	(+1.9)	0.079	0.085	(+7.6)	0.054	0.064	(+17.6)
Tb.Sp [mm]	0.557	0.571	(+2.6)	0.442	0.251	(−43.2)	0.996	0.885	(−11.2)
Ct.Th [mm]	0.94	0.91	(−3.2)	1.01	1.29	(+27.2)	0.76	0.76	(+0.7)
Biomechanical									
Scomp [N/mm]	150	147	(−1.9)	133	143	(+7.5)	70	69	(−1.0)
F.Ult [kN]	7.17	7.06	(−1.5)	6.24	6.85	(+9.8)	3.28	3.28	(+0.1)

Abbreviations: *vBMD* volumetric bone mineral density, *Dtot* total density, *Dtrab* trabecular density, *Dcort* cortical density, *BV/TV* bone to total volume, *Tb.N* trabecular number, *Tb.Th* trabecular thickness, *Tb.Sp* trabecular separation, *Ct.Th* cortical thickness, *Scomp* compression stiffness, *F.Ult* ultimate failure load

## Discussion

This study tested the effect of CFR-PEEK and two titanium VDRPs on bone density, micro-architectural and biomechanical parameters at the distal radius obtained by HRpQCT in combination with μFEA. Compared to conventional VDRPs that are made from titanium alloys, the plate made from CFR-PEEK introduced no visible image artifacts and had a minimal effect on the assessment of vBMD, micro-architectural and biomechanical parameters.

As expected, severe image artifacts were introduced by the titanium plates, which in turn led to an overestimation of the BMD, trabecular number and thickness and biomechanical properties, and an underestimation of trabecular separation at the distal radius. The errors in these bone parameters, however, differed between both titanium plates and this difference is probably related to the positioning and geometry of the plates: plate #2 was wider, thicker, and as can be seen in Fig. 3, more closely instrumented onto the radius, whereas plate #3 was smaller and a small gap between the bone and the plate was left after instrumentation. Nevertheless, the magnitude of the errors introduced by the titanium plates is higher than the reproducibility of the HRpQCT technique. Additionally, due to differences in positioning and size of the implants it seems almost impossible to correct the bone parameters in a systematic way, which makes these titanium plates unsuitable in studies when one wants to monitor bone healing with HRpQCT.

The CFR-PEEK plate, on the other hand, introduced relatively small errors that were of the same order as the reproducibility of the HRpQCT technique [8, 12, 16]. A plate made from CFR-PEEK would therefore be a suitable alternative to the conventional volar distal radius plates, if one wants to monitor the healing process of unstable distal radius fractures with HRpQCT or any other X-ray technique.

Besides the low number of subjects, a limitation of the present study is that each plate was instrumented on a different radius. However, all results were specified relative to those of the same radius without a plate, and the density and micro-architectural parameters of the radius on which the CFR-PEEK plate was instrumented was in-between those of the radii to which a titanium plate was attached, making it unlikely that this has affected the results in any way. Another important limitation of this pilot study is the difference in geometry between the three plates that were tested. It is likely that a thick, wide plate, such as plate #2, introduces more severe image artifacts and hence larger errors in bone parameters, than a thin, small plate. Furthermore, we did not test VDRPs made from stainless steel. The main reason is that at present in the Netherlands, where the study was conducted, the commonly used plates are made from titanium alloy. However, since stainless steel causes more severe image artifacts in CT than titanium [17], it is expected that stainless steel plates would lead to higher errors in HRpQCT-derived bone parameters as compared to titanium plates.

## Conclusions

In conclusion, the results of this pilot study indicate that a volar distal radius plate made from CFR-PEEK has minimal effect on bone parameters obtained at the distal radius with HRpQCT. We therefore recommend the use of CFR-PEEK plates instead of conventional titanium plates in studies that aim to monitor the healing process of distal radius fractures or bone graft ingrowth at the distal radius over time using HRpQCT.

## Abbreviations

BMD: Bone mineral density
BV/TV: Bone to total volume
CFR-PEEK: Carbon-fiber-reinforced poly-ether-ether-ketone
Ct.Th: Cortical thickness
Dcort: Cortical density
Dtot: Total density
Dtrab: Trabecular density
F.Ult: Ultimate failure load
HRpQCT: High-resolution peripheral quantitative computed tomography
MRI: Magnetic resonance imaging
RMSCV: Root-mean-square coefficient of variation
ROI: Region of interest
Scomp: Compression stiffness
Tb.N: Trabecular number
Tb.Sp: Trabecular separation
Tb.Th: Trabecular thickness
VDRP: Volar distal radius plate
